# Mike Wakelam: an appreciation

**DOI:** 10.1042/EBC20200042

**Published:** 2020-07-10

**Authors:** Bob Michell

**Affiliations:** School of Biosciences, University of Birmingham, Birmingham, U.K.

**Keywords:** autocrine pathways, lipid mediators, metabolism, paracrine pathways

## Abstract

This issue of *Essays in Biochemistry* explores lipid mediators — biologically active metabolites formed by enzymic and non-enzymic oxidation of polyunsaturated fatty acids. These can be exported across the cell membrane into the extracellular space, where they activate cell surface receptors to stimulate the cells of origin (autocrine) or nearby cells (paracrine). Lipid mediators are involved in many physiological processes, which may become dysregulated during ageing and in lipid-related diseases such as diabetes, atherosclerosis, arthritis, cancer, Alzheimer’s disease and metabolic syndrome.

Following the death in March 2020 of Professor Mike Wakelam, with the loss of his major input into the lipid signalling field, Portland Press and Guest Editors John Harwood and Emyr Lloyd-Evans decided to dedicate this issue to his memory. This Editorial briefly recalls his work and influence.

In March 2020, early in the Covid-19 pandemic, I was appalled to learn of the sudden death of my old friend and colleague Mike Wakelam (Figure 1). Mike was about to step down from a 12-year stint as director of the Babraham Research Institute just outside Cambridge, U.K., so that he could focus on laboratory research for his last few years there. At that time the Biochemical Society and Portland Press were putting together this *Lipid Mediators* collection for *Essays in Biochemistry*, and they decided that it should be dedicated to Mike’s memory. As (Sir) Pete Downes (who overlapped with Mike as a Ph.D. student in Birmingham and would later lead the University of Dundee) said of Mike: “I would emphasise his influence in three main areas: his research outputs, his mentorship and support for those who worked with him, and leadership”.

**Figure 1:**
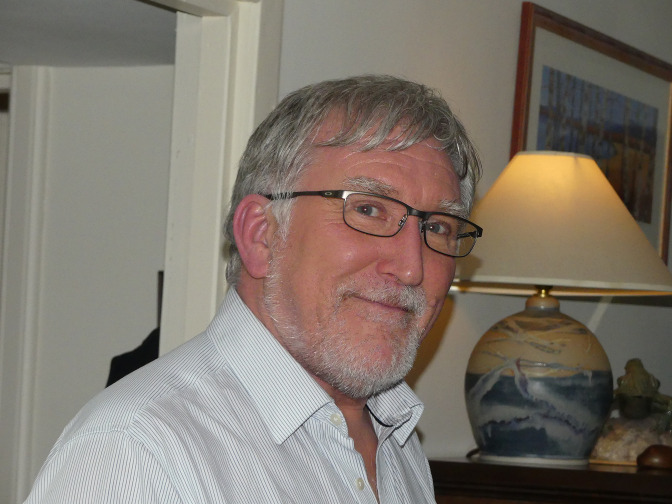
Mike at a party at Pete Downes’s home in 2018

Mike arrived in Birmingham as an outstanding Medical Biochemistry undergraduate in the mid-1970s, and stayed to do Ph.D. studies in Prof. Deryck Walker’s laboratory on the hormonal and metabolite regulation of glucokinase induction in neonatal rat hepatocytes [1]. From the start, it was obvious that he was both a first-class experimentalist and had wide interests in all of the science (and politics) around him. In particular, he became intrigued by the emerging field of phosphoinositide-based signalling that we were studying in the department’s basement. He did a little unpublished work on this, and we often discussed it in a university bar and over curries at Adil’s, which was then our favourite local balti restaurant. Soon after, he and his wife Jane Fensome – they had found each other early in their Birmingham student days – moved to Konstanz in southern Germany. The studies he did there in Dirk Pette’s laboratory, on the possible roles of lipid signalling in myoblast fusion and maturation [2], launched him into a lifetime of work on diverse aspects of lipid-mediated signalling.

Mike was recruited back to the U.K. by Miles Houslay to join the Institute of Biochemistry at Glasgow University. There, he and Miles created the Molecular Pharmacology Group to which they also recruited Graeme Milligan, and their linked labs quickly developed into one of the most vibrant and productive cell signalling research enterprises in Europe. Graeme recalled one episode when Mike was rebuked by the head of the university for wasting electricity by leaving the lights on ‘long after the lab members must have gone home’ – Mike was delighted to point out that they were still working! I have a memory of giving a seminar there that was followed by a lively evening of scientific and political discussion at the excellent ‘Ubiquitous Chip’ restaurant.

Soon after Mike got to Glasgow he and Miles, with their complementary expertises in lipid signalling and G-protein functions, collaborated with colleagues in London’s Institute of Cancer Research to show that the proto-oncogene *c*Ras was implicated in the activation of inositide-mediated growth factor signalling [3] – but it took many more years for a fuller explanation of Ras-regulated phosphoinositidase C signalling to emerge from other laboratories.

Whilst he was in Glasgow, Mike became obsessed by one of the most pervasive difficulties in lipid biochemistry: how comprehensively to analyse, with high precision, the very complex mixtures of glycerolipids (and other lipids) that are present in almost all biological samples? Before that, each group of researchers studying lipids tended to favour their own analytical variants, and it was sometimes difficult to reconcile studies from different sources. Mike and his colleagues began to address some of these challenges, initially by combining chemical modification of the lipids with sophisticated chromatographic separations [4]. He then moved back to Birmingham, to a chair in Molecular Pharmacology in our Institute of Cancer Studies. As technology advanced, he and others began to pioneer the use of user-friendly modern mass spectrometry (MS) techniques to analyse complex lipid mixtures, and ‘lipidomics’ joined the 21st century ‘-omics’ pantheon. One of the most pressing problems of those times was that multiple phosphoinositidases C and phospholipases D (PLDs) were often activated simultaneously in stimulated cells, and Mike’s group became expert in unravelling the resulting lipid confusion [4–8]. For example, his group defined key elements in the regulation and functions of mammalian PLD1 and PLD2, including that the PH domain was responsible for PtdIns(4,5)*P*_2_-dependent PLD regulation and that PLD1 and PLD2 generate structurally identical phosphatidate species that make up a highly regulated intracellular signalling pool of this lipid.

Mike had a major influence on the research directions of the Institute for Cancer Studies. Before translational research became trendy, he recognised the importance of understanding basic cell biology to better inform applied research and clinical studies. He coordinated the Institute’s computing and IT support and contributed enthusiastically to teaching, helping to develop a new M.Sc. in oncology. He recognised the need to embrace new technologies that could be applied to clinically relevant problems, and was instrumental in establishing and managing major new analytical facilities in the Medical School. He also became increasingly involved in science leadership, assessment and management, as a member of MRC Council and Chair of the MRC Molecular and Cellular Medicine Board, and by participating in many site visit assessments of research centres.

In 2007, Mike became Director of The Babraham Institute just outside Cambridge (and an Honorary Professor of Lipid Signalling at the Cambridge University Clinical School). This gave him an opportunity to lead a research institute that has been producing world-leading studies of lipids and signalling ever since the 1970s, notably by groups that through the years have been led by Rex Dawson, Robin Irvine, Mike Berridge, Phill Hawkins and Len Stephens (all FRSs). With typical energy and enthusiasm, he combined the very challenging roles of leading a large BBSRC-sponsored institute, supporting the development of the rapidly growing commercial Babraham bioscience campus, and running an internationally competitive research laboratory. In the face of major financial constraints on capital expenditure after the 2008 financial crash, his conviction that lipids are important, combined with his powers of persuasion, allowed him to create an impressive Lipidomics facility. This not only supported his own lab’s high impact science, but also facilitated work by other groups at the Institute and by the many groups elsewhere with whom he collaborated (*e.g.* [9]). Throughout his career, local collaborations would start in whichever was the communal coffee room of the time, where Mike combined his twin passions for talking about experiments and drinking coffee.

Some of this work demanded the development of novel MS methods of lipid analysis, for example to discriminate between the various rare but important phosphoinositides [10]. Mike was also one of the driving forces in creating and maintaining LIPID MAPS, a web-based international resource for lipid researchers that was initiated by Ed Dennis (University of California San Diego) and is now led by Valerie O’Donnell (University of Cardiff) [11]. In 2018, the Biochemical Society recognised his many contributions to understanding of lipid signalling by the award of their Morton Lecture, which he delivered back in Birmingham.

Despite being ambitious, Mike had an open and generous personality, and he was always aiming to help the young scientists around him to reach their potential. His great success as a mentor – to too many young colleagues to mention here – came from a mix of enthusiasm, humour, scientific insight and personal support. He became Babraham’s director under difficult financial conditions, but those around him still saw him primarily as a scientist and not as someone who had gone over to the other side. He was ever-present at seminars and student posters, asking questions and always available. He had great ambitions for the Babraham Institute and its surrounding commercial bioscience campus as a model example of how scientific curiosity and practical application could be brought together. As director, his inclusive and friendly can-do approach helped to attract many young researchers and other ambitious professionals into Babraham’s research and commercial programmes.

For all his achievements, his greatest joy and comfort was to be a husband and father. Tragically, Jane and Mike’s first child, Ellen, died in childbirth, but he is survived by Jane and their sons Alex and Patrick.

## Open Access

Open access for this article was enabled by the participation of University of Birmingham in an all-inclusive *Read & Publish* pilot with Portland Press and the Biochemical Society under a transformative agreement with JISC.

## Abbreviations

MS: mass spectrometry
PLD: phospholipase D

## References

[1] Wakelam M.J. and Walker D.G. (1981) The separate roles of glucose and insulin in the induction of glucokinase in hepatocytes isolated from neonatal rats. Biochem. J. 196, 383–390 10.1042/bj1960383 6274313 PMC1163009

[2] Wakelam M.J. and Pette D. (1984) Myoblast fusion and inositol phospholipid breakdown: causal relationship or coincidence? Ciba Found. Symp. 103, 100–118 6423350 10.1002/9780470720844.ch7

[3] Wakelam M.J., Davies S.A., Houslay M.D., McKay I., Marshall C.J. and Hall A. (1986) Normal p21N-ras couples bombesin and other growth factor receptors to inositol phosphate production. Nature 323, 173–176 10.1038/323173a0 3018591

[4] Plevin R., Cook S.J., Palmer S. and Wakelam M.J. (1991) Multiple sources of sn-1,2-diacylglycerol in platelet-derived-growth-factor-stimulated Swiss 3T3 fibroblasts. Evidence for activation of phosphoinositidase C and phosphatidylcholine-specific phospholipase D. Biochem. J. 279, 559–565 10.1042/bj2790559 1659382 PMC1151640

[5] Pettitt T.R., Martin A., Horton T., Liossis C., Lord J.M. and Wakelam M.J. (1997) Diacylglycerol and phosphatidate generated by phospholipases C and D, respectively, have distinct fatty acid compositions and functions. Phospholipase D-derived diacylglycerol does not activate protein kinase C in porcine aortic endothelial cells. J. Biol. Chem. 272, 17354–17359 10.1074/jbc.272.28.17354 9211874

[6] Hodgkin M.N., Pettitt T.R., Martin A., Michell R.H., Pemberton A.J. and Wakelam M.J. (1998) Diacylglycerols and phosphatidates: which molecular species are intracellular messengers? Trends Biochem. Sci. 23, 200–204 10.1016/S0968-0004(98)01200-6 9644971

[7] Pettitt T.R. and Wakelam M.J. (1999) Diacylglycerol kinase epsilon, but not zeta, selectively removes polyunsaturated diacylglycerol, inducing altered protein kinase C distribution *in vivo*. J. Biol. Chem. 274, 36181–36186 10.1074/jbc.274.51.36181 10593903

[8] McDermott M.I., Wang Y., Wakelam M.J.O. and Bankaitis V.A. (2020) Mammalian phospholipase D: function, and therapeutics. Prog. Lipid Res. 78, 101018 10.1016/j.plipres.2019.101018 31830503 PMC7233427

[9] Farquhar M.J., Humphreys I.S., Rudge S.A., Wilson G.K., Bhattacharya B., Ciaccia M. et al. (2017) Autotaxin-lysophosphatidic acid receptor signalling regulates hepatitis C virus replication. J. Hepatol. 66, 919–929 10.1016/j.jhep.2017.01.009 28126468

[10] Wakelam M.J. and Clark J. (2011) Methods for analyzing phosphoinositides using mass spectrometry. Biochim. Biophys. Acta 1811, 758–762 10.1016/j.bbalip.2011.09.004 21964281

[11] O’Donnell V.B., Dennis E.A., Wakelam M.J.O. and Subramaniam S. (2019) LIPID MAPS: serving the next generation of lipid researchers with tools, resources, data, and training. Sci. Signal. 12, eaaw2964 10.1126/scisignal.aaw2964 30622195

